# Supplementary material for: Radiolytically reworked Archean organic matter in a habitable deep ancient high-temperature brine
Source: Nat Commun. 2023 Oct 3;14:6163. doi: 10.1038/s41467-023-41900-8 (PMC10547683; doi:10.1038/s41467-023-41900-8)
Supplement: Supplementary file 1 — Supplementary Information [file 41467_2023_41900_MOESM1_ESM.pdf]

Supplementary Information

Radiolytically reworked Archean organic matter in a habitable deep ancient high-temperature brine

Devan M. Nisson[1*], Clifford C. Walters[2], Martha L. Chacón-Patiño[3], Chad R. Weisbrod[3], Thomas L. Kieft[4], Barbara Sherwood Lollar[5,6], Oliver Warr[7], Julio Castillo[8], Scott M. Perl[9], Errol D. Cason[10], Barry M. Freifeld[11], Tullis C. Onstott[1]

[1] Department of Geosciences, Princeton University, Princeton, NJ 08540, USA

[2] Bureau of Economic Geology, University of Texas, Austin, TX 78758, USA

[3] National High Magnetic Field Laboratory, Tallahassee, FL 32310 USA

[4] Department of Biology, New Mexico Institute of Mining and Technology, Socorro, NM 87801, USA

[5] Department of Earth Sciences, University of Toronto, Toronto, Ontario M5S 3B1, Canada

[6] Institut de Physique du Globe de Paris (IPGP), Université Paris Cité, France

[7] Department of Earth Sciences, University of Ottawa, Ottawa, Ontario K1N 6N5, Canada

[8] Department of Microbiology and Biochemistry, University of the Free State, Bloemfontein 9300, South Africa

[9] NASA Jet Propulsion Laboratory, California Institute of Technology, Pasadena, CA 91109, USA

[10] Department of Animal Sciences, University of the Free State, Bloemfontein 9300, South Africa

[11] Lawrence Berkeley National Laboratory, Berkeley, CA 94720, USA

*Corresponding Author Email: dnisson@princeton.edu

1. Supporting Tables and Figures

Table S1. Composition (%) with carbon and hydrogen isotopic values (‰) for C_{1-4} hydrocarbon gases.

Sampling Level*	CH_4 (%)	$\delta^{13}C$ (‰)- CH_4	δ^2H (‰)- CH_4	C_2H_6 (%)	$\delta^{13}C$ (‰)- C_2H_6	δ^2H (‰)- C_2H_6	C_3H_8 (%)	$\delta^{13}C$ (‰)- C_3H_8	δ^2H (‰)- C_3H_8	C_4H_{10} (%)**	$\delta^{13}C$ (‰)- C_4H_{10}***	δ^2H (‰)- C_4H_{10}***	C_1/C_{2+}
95-level	44.92	-41.0	-412	5.88	-40.5	-285	0.41	-36.2	-198	b.d.	b.d.	b.d.	7.1
101-level	26.40	-39.7	-361	0.79	-41.0	-208	b.d.	b.d.	b.d.	b.d.	b.d.	b.d.	33.4
DR548	50.7	-46.5	-403	3.85	-50.5	-285	0.52	-47.2	-192	0.11	-45.6	-72	11.3
MP104	49.6	-32.8	-366	3.99	-37.6	-270	0.60	-34.8	-193	0.13	-35.3	-88	10.5
KL739	64.9	-28.7	-300	2.86	-30.7	-230	0.41	-27.2	-142	0.08	b.d.	b.d.	19.3
CCS4546	69.5	-32.0	-452	7.14	-34.6	-348	0.78	-33.4	b.d.	0.17	b.d.	b.d.	8.6
KC7792	75.7	-38.3	-390	10.0	-38.2	-299	1.66	-37.6	-256	0.48	-37.9	-224	6.2

b.d. = below detection

*Values for sample KC7792 are from Sherwood Lollar et al. [1]. Values for samples DR549, MP104, KL739, and CCS4546 are from Sherwood Lollar et al. [2].

**C_4H_{10} (%) incorporates both n-butane and iso-butane for all samples

***$\delta^{13}C$ (‰)- C_4H_{10} and δ^2H (‰)- C_4H_{10} of sample KL739 includes iso-butane with n-butane (all others based on n-butane)

Table S2. Light element abundance and composition for the Moab Khotsong system, as well as their associated mass stopping powers and neutron yields for U and Th from Andrews et al. [3] Table 2.

	µg/g (Composition in Moab Khotsong)	Fractional abundance	Mass Stopping Powers (MeV g^{-1} cm^{-2})	Neutron Yield per µg of ^{238}U (g^{-1} a^{-1})	Neutron Yield per µg of ^{232}Th (g^{-1} a^{-1})
O	1,886,500	0.615	609	0.24	0.08
F	440	0.000	531	31.6	12.5
Na	60,000	0.020	503	12.5	5.75
Mg	60,000	0.020	520	5.52	2.33
Al	260,000	0.085	493	4.88	2.39
Si	500,000	0.163	501	0.68	0.32
K	8,000	0.003	455	0.37	0.09

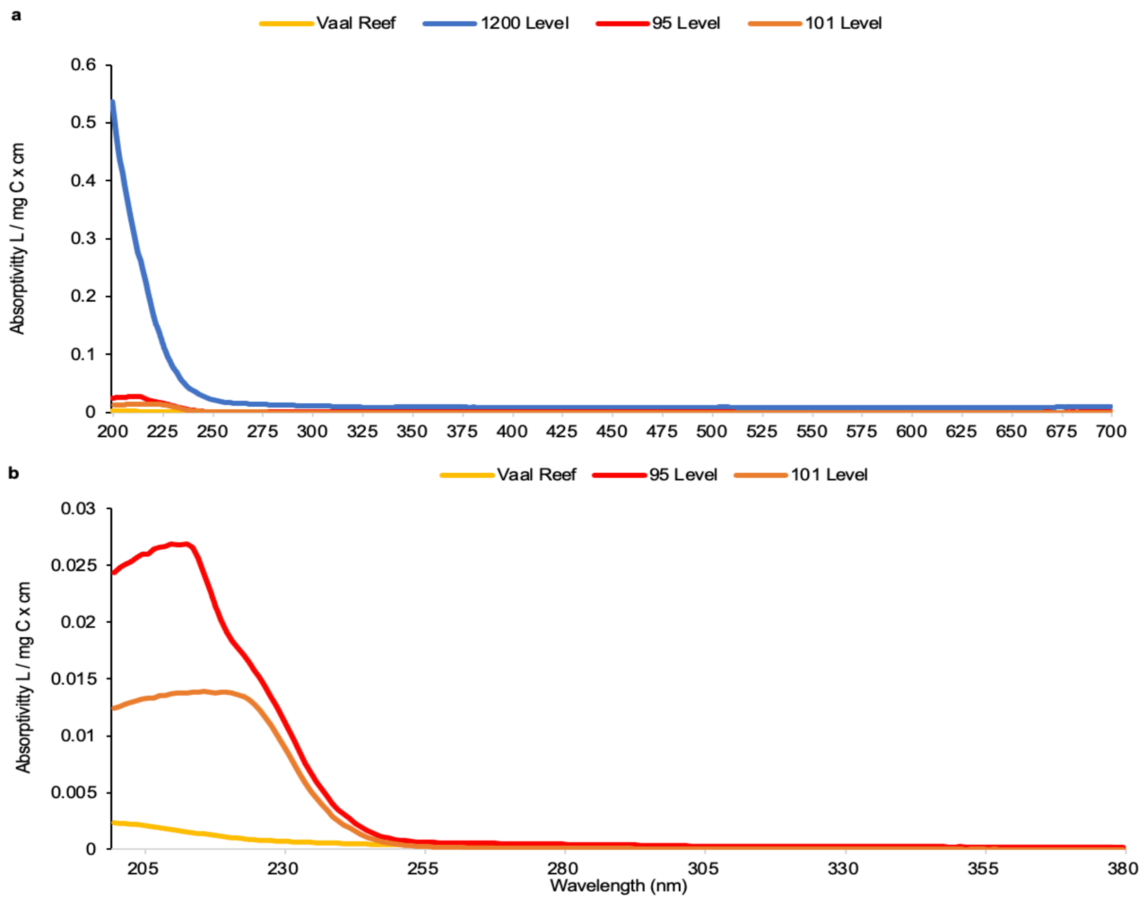

Fig. S1. UV-Vis absorptivity of Moab Khotsong fluids. (a) Absorptivity vs. wavelength (200-700 nm) for Moab Khotsong fracture fluids and Vaal Reef sample. (b) Closer view of absorptivity vs. wavelength (200-380 nm) for Moab Khotsong brines and Vaal Reef Sample. UV-Vis spectra of both 95 and 101-level brines showed decreasing absorptivity with increasing wavelength, with no measurable absorptivity at wavelengths >250nm. Absorptivity values covered a range up to 0.03 L/(mg C · cm) between 200 and 240 nm, highlighting the presence of different organic species present in the brine DOC.

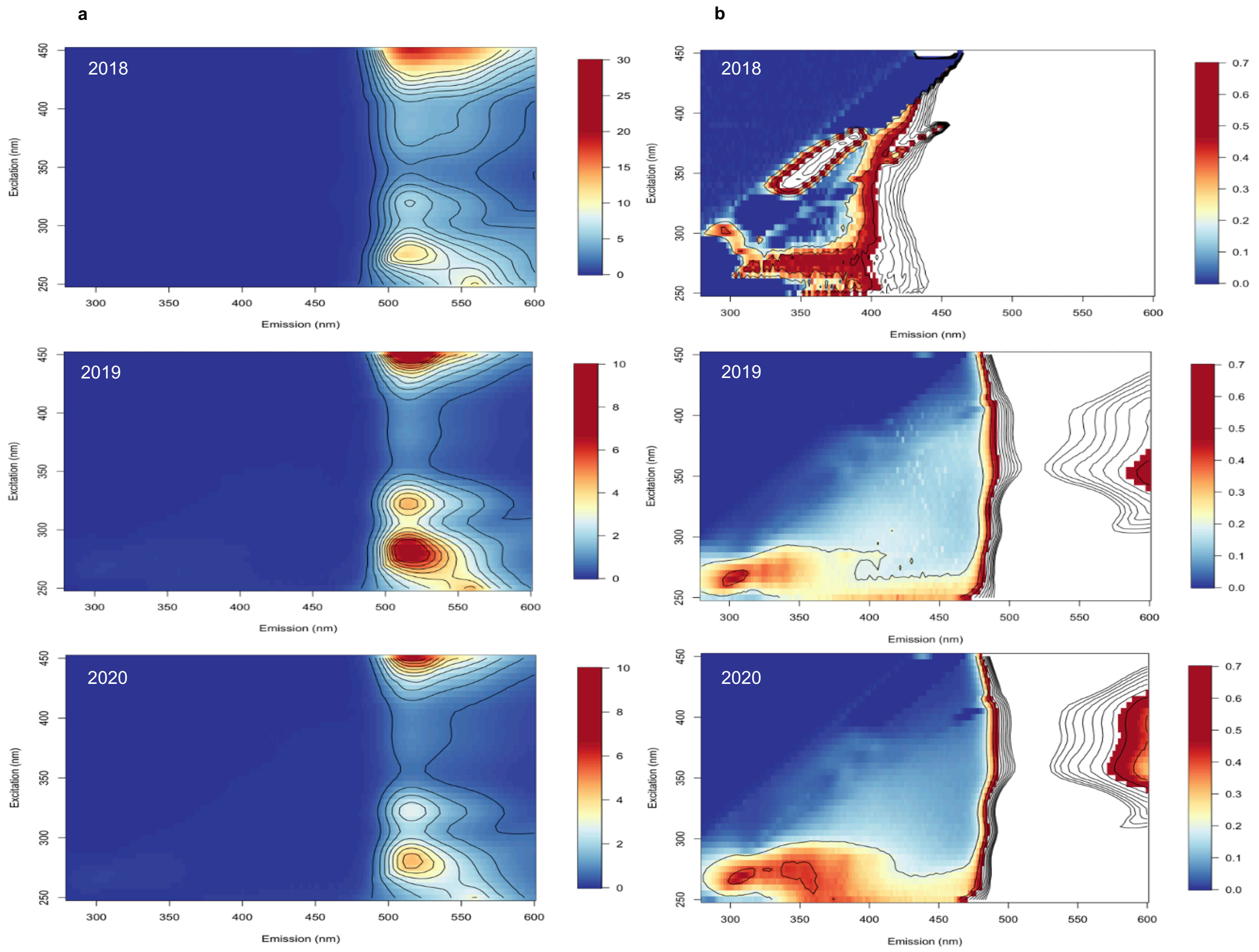

Fig. S2. Excitation-Emission Matrices (EEMs) for 95-level brine sampled in 2018, 2019, and 2020. Spectra (a) with fluorescein, and (b) without fluorescein signature included. Scales represent fluorescence intensity. Contour lines for (a) are 0, 1, 10 for all samples. Contour lines for (b) are 0, 0.001, 0.01 for 2018 and 0, 0.05, 0.5 for 2019 and 2020 samples. The 95-level brine displayed a dominating fluorescence signature from the fluorescein dye throughout all λ_{em} ranging from ~ 500 to 600 nm. Comparing 95-level DOC samples over time, there is a clear reduction in the fluorescein signal from 2018 to 2019, with a continued decrease into 2020. This provides additional evidence of borehole flushing and decreasing potential contamination from drilling fluid over the three-year sampling period for this sample.

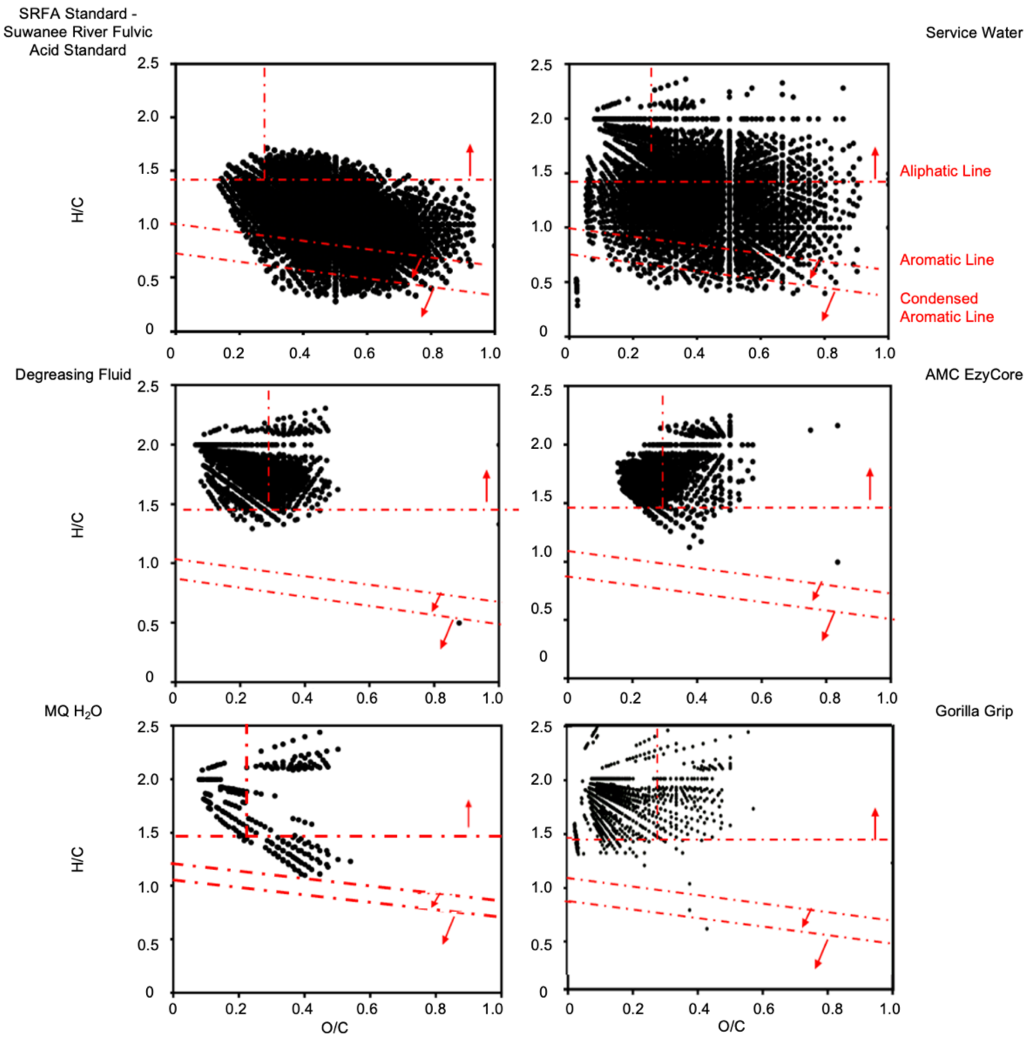

Fig. S3. Van Krevelen diagrams from negative ion ESI 21 tesla FT-ICR MS of service water and organic additives used during borehole drilling. Samples shown include: Degreasing Fluid, AMC-EzyCore, and Gorilla Grip. A SRFA standard is included as an example of fulvic acid from modern terrestrial plant matter, alongside an MQ H_2O procedural blank to account for contaminants introduced during solid phase extraction. Dashed red lines and arrows indicate regions of increasing (1) aliphatic, (2) aromatic, and (3) condensed aromaticity of molecules. FT-ICR MS spectra for samples shown are included in Supplementary Data 1.

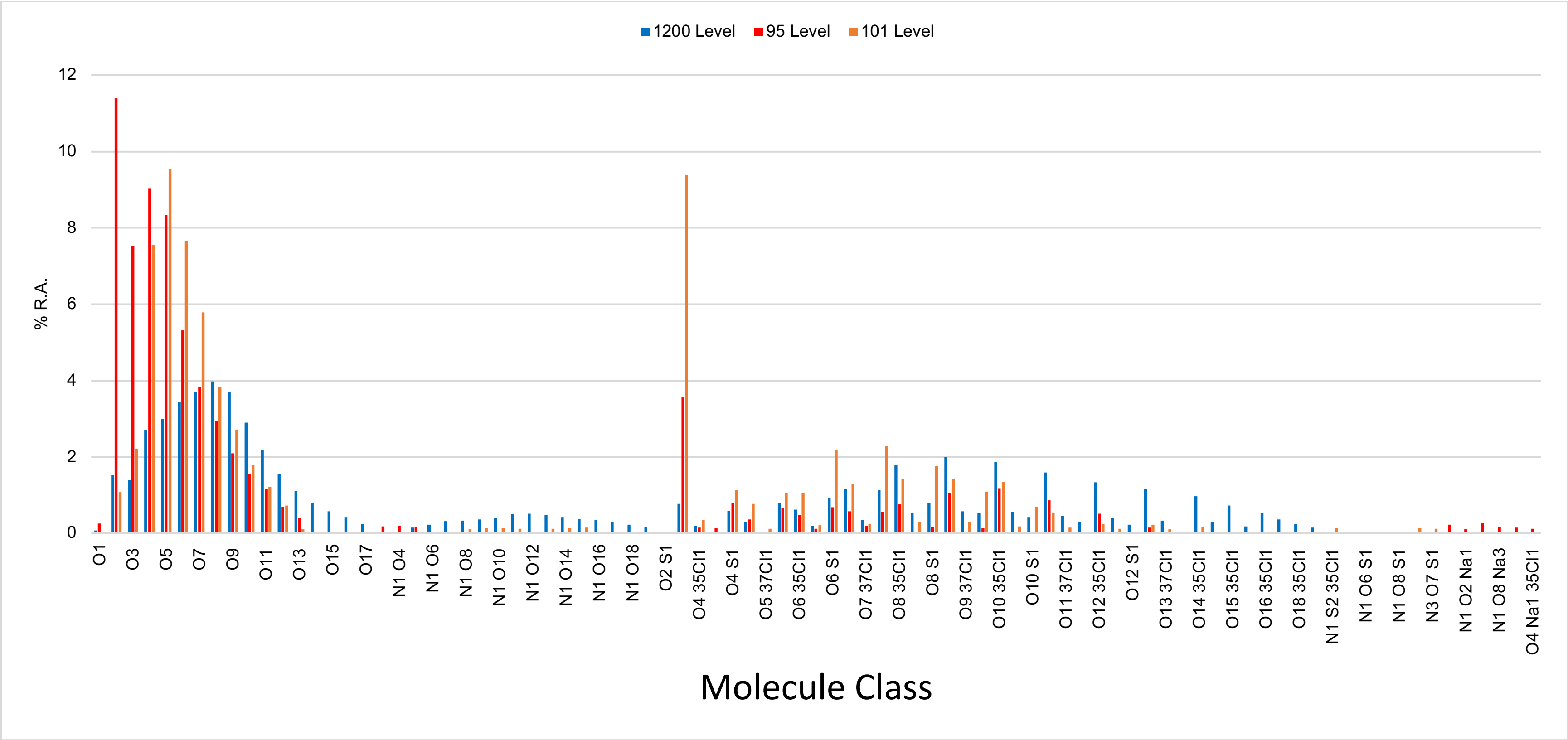

Fig. S4. Percent relative abundance (%R.A.) of organic species. Includes all heteroatom classes identified through negative ion ESI 21 tesla FT-ICR MS for 95 and 101-level brines compared to the 1200-level fluid. FT-ICR MS spectra for samples shown are included in Supplementary Data 1.

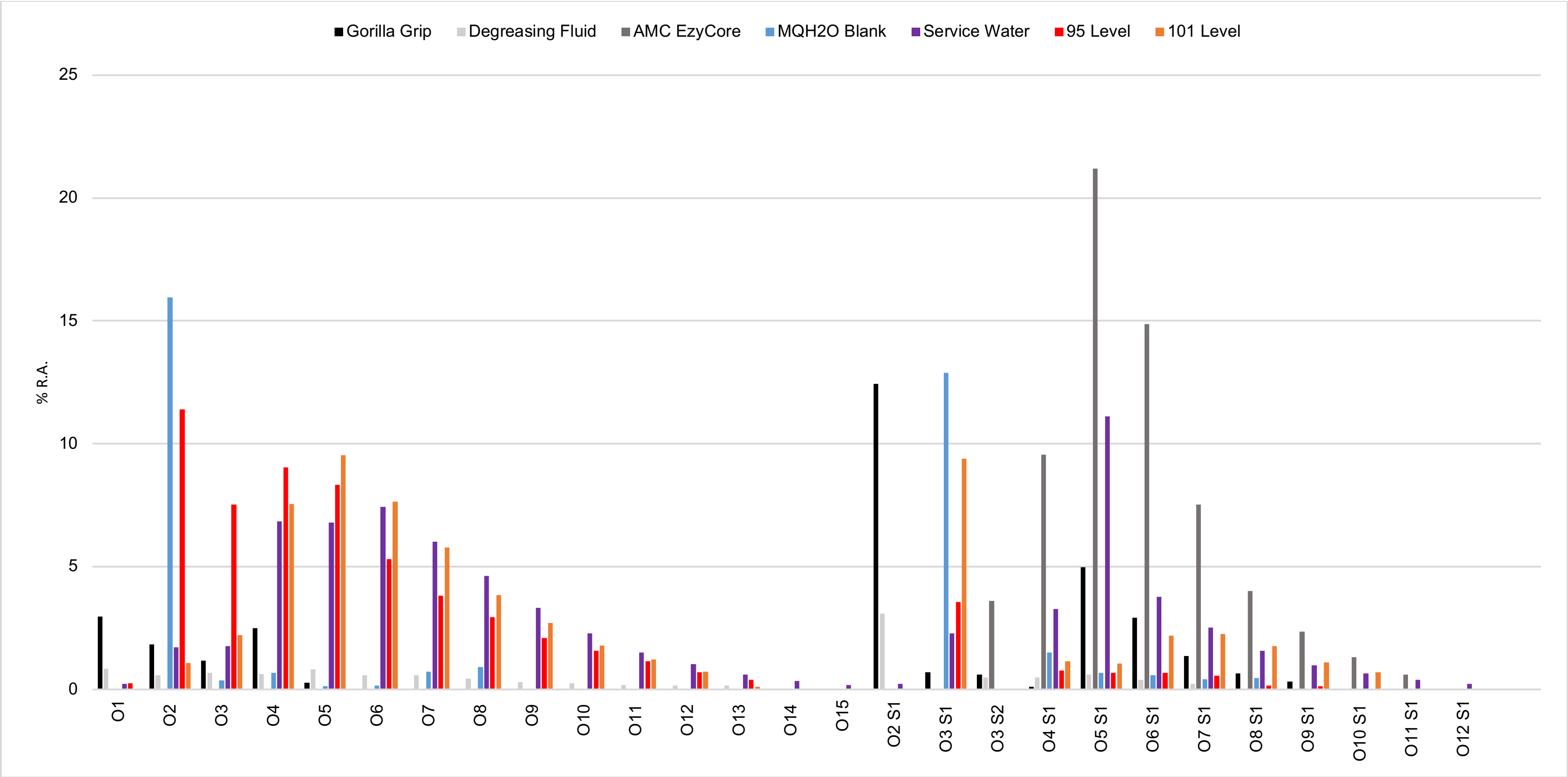

Fig. S5. Percent relative abundance (%R.A.) of organic species in major classes O_x, N_1O_x, and SO_x. Classifications obtained through negative ion ESI 21 tesla FT-ICR MS for 95 and 101-level brines compared to drilling additives and an MQ H_2O Blank. If normalized for total sample DOC concentration, %R.A. peaks for MQ H_2O (~1 mg C/L total DOC) would not be visible next to brine peaks. FT-ICR MS spectra for samples shown are included in Supplementary Data 1.

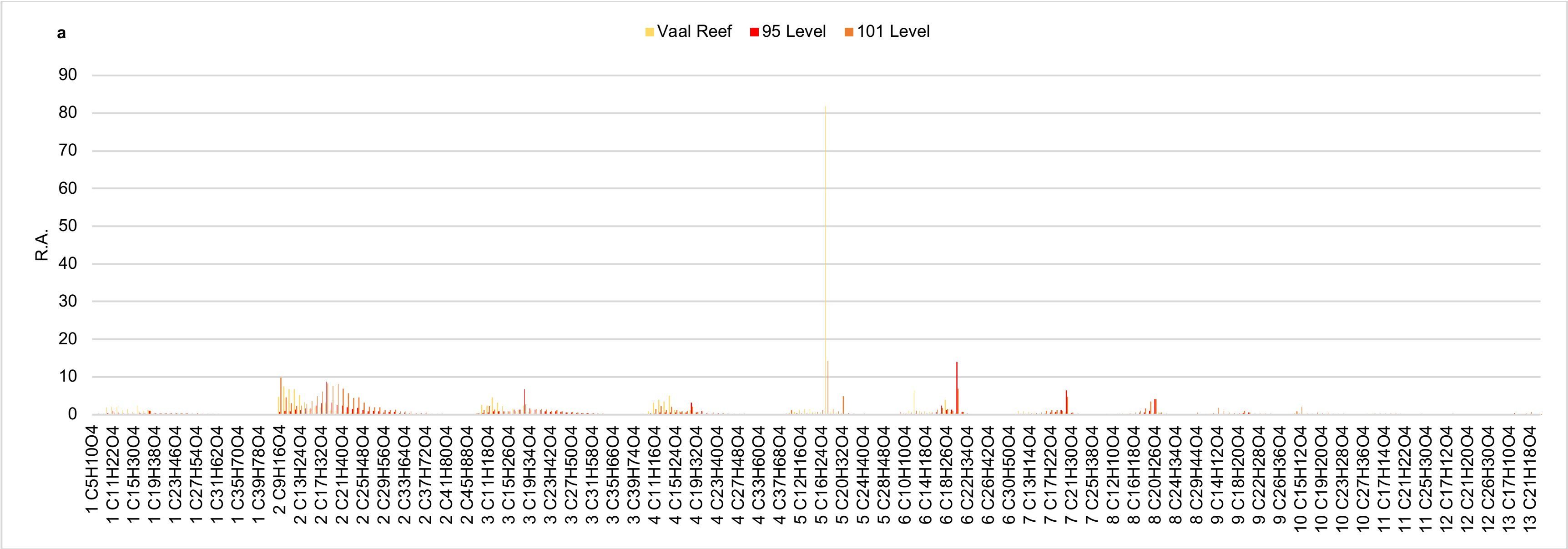
a
Vaal Reef
95 Level
101 Level
R.A.
90
80
70
60
50
40
30
20
10
0
1 C5H10O4
1 C11H22O4
1 C15H30O4
1 C19H38O4
1 C23H46O4
1 C27H54O4
1 C31H62O4
1 C35H70O4
1 C39H78O4
2 C9H16O4
2 C13H24O4
2 C17H32O4
2 C21H40O4
2 C25H48O4
2 C29H56O4
2 C33H64O4
2 C37H72O4
2 C41H80O4
2 C45H88O4
3 C11H18O4
3 C15H26O4
3 C19H34O4
3 C23H42O4
3 C27H50O4
3 C31H58O4
3 C35H66O4
3 C39H74O4
4 C11H16O4
4 C15H24O4
4 C19H32O4
4 C23H40O4
4 C27H48O4
4 C33H60O4
4 C37H68O4
5 C12H16O4
5 C16H24O4
5 C20H32O4
5 C24H40O4
5 C28H48O4
6 C10H10O4
6 C14H18O4
6 C18H26O4
6 C22H34O4
6 C26H42O4
6 C30H50O4
7 C13H14O4
7 C17H22O4
7 C21H30O4
7 C25H38O4
8 C12H10O4
8 C16H18O4
8 C20H26O4
8 C24H34O4
8 C29H44O4
9 C14H12O4
9 C18H20O4
9 C22H28O4
9 C26H36O4
10 C15H12O4
10 C19H20O4
10 C23H28O4
10 C27H36O4
11 C17H14O4
11 C21H22O4
11 C25H30O4
12 C17H12O4
12 C21H20O4
12 C26H30O4
13 C17H10O4
13 C21H18O4

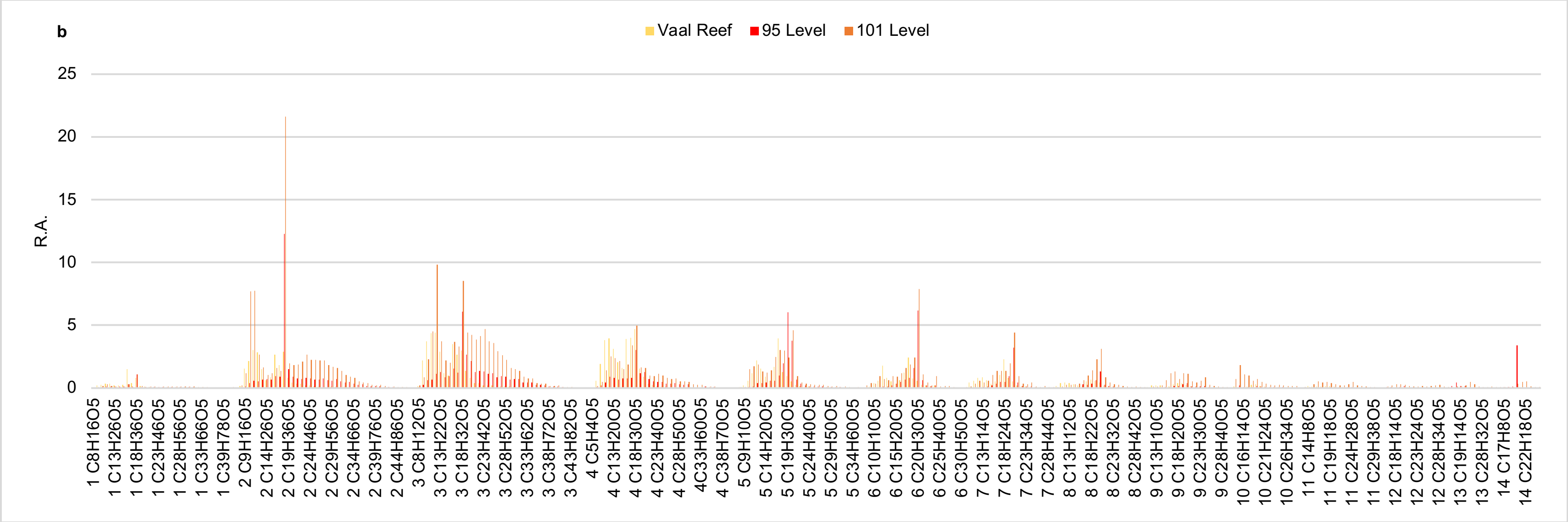
b
Vaal Reef
95 Level
101 Level
R.A.
25
20
15
10
5
0
1 C8H16O5
1 C13H26O5
1 C18H36O5
1 C23H46O5
1 C28H56O5
1 C33H66O5
1 C39H78O5
2 C9H16O5
2 C14H26O5
2 C19H36O5
2 C24H46O5
2 C29H56O5
2 C34H66O5
2 C39H76O5
2 C44H86O5
3 C8H12O5
3 C13H22O5
3 C18H32O5
3 C23H42O5
3 C28H52O5
3 C33H62O5
3 C38H72O5
3 C43H82O5
4 C5H4O5
4 C13H20O5
4 C18H30O5
4 C23H40O5
4 C28H50O5
4C33H60O5
4 C38H70O5
5 C9H10O5
5 C14H20O5
5 C19H30O5
5 C24H40O5
5 C29H50O5
5 C34H60O5
6 C10H10O5
6 C15H20O5
6 C20H30O5
6 C25H40O5
6 C30H50O5
7 C13H14O5
7 C18H24O5
7 C23H34O5
7 C28H44O5
8 C13H12O5
8 C18H22O5
8 C23H32O5
8 C28H42O5
9 C13H10O5
9 C18H20O5
9 C23H30O5
9 C28H40O5
10 C16H14O5
10 C21H24O5
10 C26H34O5
11 C14H8O5
11 C19H18O5
11 C24H28O5
11 C29H38O5
12 C18H14O5
12 C23H24O5
12 C28H34O5
13 C19H14O5
13 C28H32O5
14 C17H8O5
14 C22H18O5

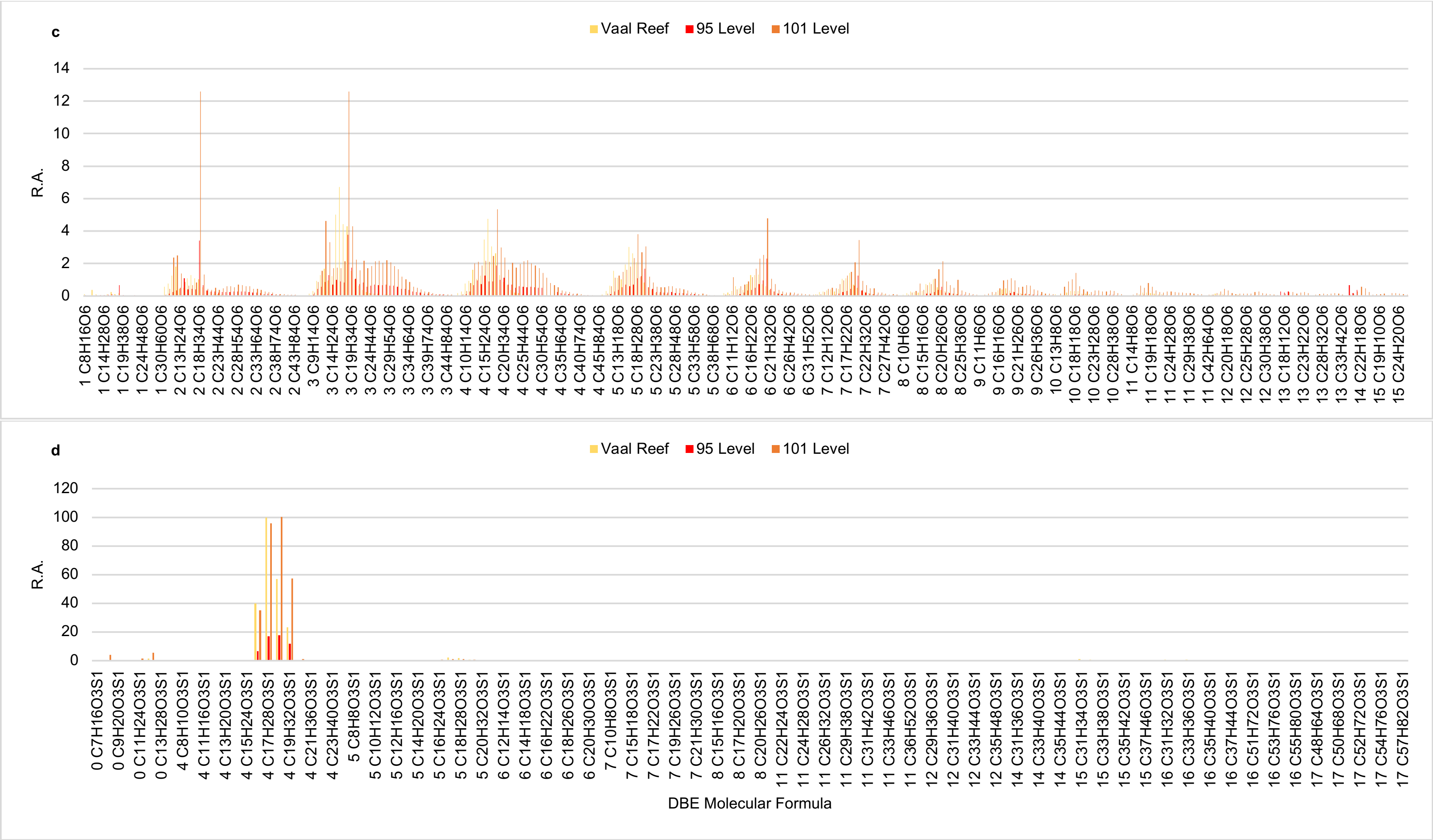

Fig. S6. Relative abundance of organic species from negative ion ESI 21 tesla FT-ICR MS in 95 and 101-level brines vs. Vaal Reef. Includes (a) O_4, (b) O_5, (c) O_6, and (d) S_1O_3 classes. Terms on the X-axis consist of the DBE number followed by molecular formula assigned to a given peak. The large peak at $C_{17}H_{26}O_4$ for the Vaal Reef in (a) is a noise artifact and not an accurate representation of the sample composition. FT-ICR MS spectra for samples shown are included in Supplementary Data 1.

2. ^{14}C Production in Subsurface Fluids

Pathways of ^{14}C Contribution to the Moab Khotsong Brines

Here we explore three major cases to explain ^{14}C introduction into noble-gas labeled 'ancient' fracture waters. Cases (1) and (2) are explored with equations of Heard et al. [4] for Wits Basin fracture waters. Case 3 presents a more in depth look at radiolytic contribution based on the texts of Andrews et al., [3] and Andrews et al., [5].

Case 1: Contamination from air introduction during sampling:

Estimate of the relative volumetric rate of air addition to the system:

$$F_{air} = F_{gas} \cdot X_{air} \qquad (1)$$

Where F_{air} is the flow rate of contaminating air (L/min), F_{gas} is the flow rate of groundwater-exsolved gas (L/min), and *X* is the fraction of air contamination (ranges from 1-3% based on air contamination from Warr et al. [6]).

F_{gas} = 0.020 L/min [measured value at 95-level]
X_{air} = 3%
F_{air} = 0.00060 L/min

Calculate the number of moles of CO_2 per liter of air:

$$M_{CO_2}^{air} = 4.0 \cdot 10^{-4}/(R \cdot T) = 1.5 \cdot 10^{-5} \qquad (2)$$

R = gas constant (8.2 ×10^{-2} (L · atm)/K/mol)
T = temperature in K (in our case for 95-level, 54°C or 327K)
$4.0 \cdot 10^{-4}$ is the partial pressure of CO_2 in air (in atm)

Calculate the moles per liter addition of modern CO_2 to sample groundwater:

$$M_{CO_2} = (F_{air} \cdot M_{CO_2}^{air})/F_W = 8.00 \cdot 10^{-8} \qquad (3)$$

$F_W = water\ flow\ rate$ = 0.112 L/min for 95-level
*F_{air} value used here was 0.00060 L/min (upper contamination estimate)

The amount of added CO_2 that will dissolve into the sample water and be recorded as DIC:

$M_{CO_2}^{dis} = M_{CO_2} \cdot (1 - (\frac{22400 \cdot \rho_W \cdot T \cdot F_W}{1000 \cdot 273 \cdot K_{CO_2}^{m} \cdot F_{air}} + 1)^{-1})$ (4)

ρ_W = density of fluid in g/cm^3 = 1.04 g/cm^3 for the 95-level brine

$K_{CO_2}^{m}$=Henry's constant for CO_2 in molar [3.4 · 10^{-2} mol / (L · atm)]

$M_{CO_2}^{dis}$=7.96 · 10^{-8} mol/L or 7.96 * 10^{-5} mmol/L→ doesn't compare to concentrations of 0.266 or 1.07 mmol/L DIC for the 95 or 101-levels

Conclusion: Air contamination during sample is likely only a minor contributor to the ^{14}C content of the brines. *See note for DIC ages at the bottom of this section.

*The DIC ages of the Moab Khotsong brines were based on ^{14}C measurements taken on several year-old fluid samples collected in glass NOSAM bottles with greased stoppered caps. Over this time the grease likely dried out (based on analysis of other old Witwatersrand fluid samples stored in the same way), leading to younger ^{14}C based ages not captured in calculations of air contamination (Case 1), as those are dependent on gas and water flow rates at site.

<u>Case 2: Contamination from mixing with a modern, surface-derived fluid:</u>

Consider an end-member mixing scenario where a young water component contains essentially modern ^{14}C and the DIC concentrations in both components are assumed to be equal and dominated by atmospheric recharge.

Using estimated ages from measured $\Delta^{14}C$:

95-level: 9,530 = -8033$\ln(F_m)$ where F_m= 30% contribution from modern fluid with modern ^{14}C

101-level: 8,250 =-8033$\ln(F_m)$ where F_m= 36% contribution from modern fluid with modern ^{14}C

1200-level: 6,410 = -8033$\ln(F_m)$ where F_m= (up to) 45% contribution from modern fluid with modern ^{14}C

If this contribution from young water scales to contribution on noble-gas estimated residence times, this would result in an underestimate of the 95-level closed-system residence time by 30Ma. This can be accounted for in the error on noble gas derived residence times for this fluid system ($\pm$0.14 – 1.87 Ga; Warr et al. [6]).

Problems with modern water mixing contribution:
We might expect up to 36% contribution from modern fluid mixing in the brines to push their δ^2H and δ^{18}O stable water isotope signatures closer to the meteoric waterline (especially since the 1200-level has up to 45% modern water contribution, and does actually lie close to the meteoric water line for δ^2H and δ^{18}O (Warr et al. [7]). The same argument could be made for salinity, unless we have modern mixing with an extremely ancient and salty end-member (more so than what the current brine signatures suggest (Nisson et al. [8])), causing the brine values to still appear very old and salty. This is unlikely as it would require a more isolated end-member brine than has ever been previously characterized.

Conclusions: Mixing with a younger fluid is possible given the error on noble gas-derived age estimates for the 95-level brine. Because the 101 and 95-level brine estimated DIC ages based on ^{14}C are similar, there isn't evidence to suggest that one brine mixed with younger water to a greater extent than the other. Younger water mixing is not reflected in the stable isotope or salinity parameters for these brines, suggesting that mixing with younger waters cannot be ruled out as a possible contributor of ^{14}C, but significant mixing is not supported by the geochemistry or isotopic composition of the brines.

Contribution of ^{14}C from in-situ radionuclide decay:

To determine the contribution of natural radionuclide decay to in situ ^{14}C production, the neutron flux in the Moab brine was calculated based on equations (1), (2) and (3) in Andrews et al. [3] as well as equations (8) and (14) in Andrews et al. [5]:

$$P_n = {}^{238}N\lambda_{sf}\upsilon + a[U] + b[Th]s^{-1}g^{-1} \qquad (1)$$

Where P_n = Neutron production rate, ^{238}N = number of ^{238}U atoms/g, λ_{sf} = spontaneous fission decay constant for ^{238}U ($8.5 \cdot 10^{-17}$ a^{-1}; De Carvalho et al. [9]), υ = average number of neutrons emitted per spontaneous fission of ^{238}U (2.2)[10], and [U] and [Th] concentrations in ppm (each estimated at 100 ppm following radiolytic brine formation estimates in Nisson et al. [8]).

Factors a and b concern fractional abundance of light elements in the system. Those used for the Moab system are included in Table S2 in Appendix I.

$$a = \frac{\sum_1^i S_i F_i Y_i^U}{\sum_1^i S_i F_i} \qquad (2)$$

$$b = \frac{\sum_1^i S_i F_i Y_i^{Th}}{\sum_1^i S_i F_i} \qquad (3)$$

Where S_i = Mass stopping power (6.8 MeV), F_i = the fractional abundance for light element *i* (Table S2), and Y_i^U and Y_i^{Th} are the neutron yields per µg/g of U and Th in equilibrium with their daughters.

$$n_t = \frac{P_n}{K}\ when\ t > 10^3 s \qquad (8)$$

Where $K = \sigma_m \cdot 220{,}000$ [σ_m is the weighted mean absorption cross section, estimated at 0.0066 $cm^2\ g^{-1}$]. n_t multiplied by the velocity of a neutron (220,000 cm s^{-1}) results in neutron flux (ϕ) $cm^{-2}\ s^{-1}$ for the system. In the case of Moab, this value was $9.53 \cdot 10^{-4}\ cm^{-2}\ s^{-1}$.

Decay of ^{14}N and ^{17}O were considered as the primary ^{14}C producing reactions in the Moab brine:
$^{14}N + n \Rightarrow {}^{14}C + p$ (reaction cross section $1.86 \cdot 10^{-24}\ cm^2$ from Andrews et al. [3])
$^{17}O + n \Rightarrow {}^{14}C + a$ (reaction cross section $2.45 \cdot 10^{-25}\ cm^2$ from Andrews et al. [3])

$$^{14}C = \frac{\sigma N \phi}{\lambda_r}(1 - e^{-\lambda_r t}) \qquad (14)$$

Where σ = cross section for the reaction considered, N = amount of parent species in atoms / cm^3, λ_r = decay constant of ^{14}C ($3.80 \cdot 10^{12}$ s), and t = time in s.
Using estimates of $3.47 \cdot 10^{18}$ (^{14}N atoms / cm^3) and $2.67 \cdot 10^{19}$ (^{17}O atoms / cm^3) and, the total atomic ^{14}C production equaled $3.26 \cdot 10^3$ atoms cm^{-3}.
Over the lifetime of the 1.2 Ga brine (assuming a closed-system), this production may account up to 7.5% modern ^{14}C. This value could be larger if the system receives more radionuclide contribution from the reef than estimated here.

Conclusions: There is likely significant *in situ* radiolytic contribution to ^{14}C in the Moab brines, explaining most of the age discrepancy between ^{14}C ages and noble gas estimated residence times for this system. This is due to contribution from the radiogenic rich gold reef in this system, and is supported by high noble gas radiogenic excesses, including the highest ever ^{86}Kr radiogenic excess found in subsurface fracture fluids (Warr et al. [6]).

Supplementary References

1. Sherwood Lollar, B., Westgate, T.D., Ward, J.A., Slater, G.F. & Lacrampe-Couloume, G. Abiogenic formation of alkanes in the Earth's crust as a minor source for global hydrocarbon reservoirs. *Nature* **416**, 522-524 (2002).
2. Sherwood Lollar, B., Lacrampe-Couloume, G., Slater, G.F., Ward, J., Moser, D.P., Gihring, T.M., Lin, L.H. & Onstott, T.C. Unravelling abiogenic and biogenic sources of methane in the Earth's deep subsurface. *Chem. Geol.* **226**, 328-339 (2006).
3. Andrews, J.N., Davis, S.N., Fabryka-Martin, J., Fontes, J.C., Lehmann, B.E., Loosli, H.H., Michelot, J.L., Moser, H., Smith, B. & Wolf, M. The *in situ* production of radioisotopes in rock matrices with particular reference to the Stripa granite. *Geochim. Cosmochim. Acta.* **53**, 1803-1815 (1989).
4. Heard, A.W., Warr, O., Borgonie, G., Linage, B., Kuloyo, O., Fellowes, J.W., Magnabosco, C., Lau, M.C., Erasmus, M., Cason, E.D., van Heerden, E., Kieft, T.L., Mabry, J.C., Onstott, T.C., Sherwood Lollar, B. & Ballentine, C.J. South African crustal fracture fluids preserve paleometeoric water signatures for up to tens of millions of years. *Chem. Geol.* **493**, 379-395 (2018).
5. Andrews, J.N., Fontes, J.C., Michelot, J.L. & Elmore, D. In-situ neutron flux, ^{36}Cl production and groundwater evolution in crystalline rocks at Stripa, Sweden. *Earth Planet. Sci. Lett.* **77**, 49-58 (1986).
6. Warr, O., Ballentine, C.J., Onstott, T.C., Nisson, D.M., Kieft, T.L., Hillegonds, D.J. & Sherwood Lollar, B. ^{86}Kr excess and other noble gases identify a billion-year-old radiogenically-enriched groundwater system. *Nat. Commun.* **13**, 3768 (2022).
7. Warr, O., Giunta, T., Onstott, T.C., Kieft, T.L., Harris, R.L., Nisson, D.M. & Sherwood Lollar, B. The role of low-temperature ^{18}O exchange in the isotopic evolution of deep subsurface fluids. *Chem. Geol.* **561**, 120027 (2021).
8. Nisson, D.M., Kieft, T.L., Drake, H., Warr, O., Sherwood Lollar, B., Ogasawara, H., Perl, S.M., Friefeld, B.M., Castillo, J., Whitehouse, M.J., Kooijman, E. & Onstott, T.C. Hydrogeochemical and isotopic signatures elucidate deep subsurface hypersaline brine formation through radiolysis driven water-rock interaction. *Geochim. Cosmochim. Acta.* **340**, 65-84 (2023).
9. De Carvalho, H.G., Martins, J.B., Medeiros, E.L. and Tavares, O.A.P. Decay constant for the spontaneous-fission process in 238U. *Nucl. Instrum. Methods Phys. Res.* **197,** 417-426 (1982).
10. U.E.A.E.C. Reactor Physics Constants. Argonne National Laboratory Report **ANL-5800**, 850p (1963).
