# Supplementary material for: Radiolytically reworked Archean organic matter in a habitable deep ancient high-temperature brine
Source: Nat Commun. 2023 Oct 3;14:6163. doi: 10.1038/s41467-023-41900-8 (PMC10547683; doi:10.1038/s41467-023-41900-8)
Supplement: Supplementary file 3 — Description of Additional Supplementary Files [file 41467_2023_41900_MOESM3_ESM.pdf]

Description of Additional Supplementary Files:

Supplementary Data 1: This zip file contains source data for FT-ICR MS spectra included in this manuscript.
