# Supplementary material for: Radiolytically reworked Archean organic matter in a habitable deep ancient high-temperature brine
Source: Nat Commun. 2023 Oct 3;14:6163. doi: 10.1038/s41467-023-41900-8 (PMC10547683; doi:10.1038/s41467-023-41900-8)
Supplement: Supplementary file 5 — Reporting Summary [file 41467_2023_41900_MOESM5_ESM.pdf]

nature portfolio

Corresponding author(s): Devan Nisson

Last updated by author(s): 09_08_23

Reporting Summary

Nature Portfolio wishes to improve the reproducibility of the work that we publish. This form provides structure for consistency and transparency in reporting. For further information on Nature Portfolio policies, see our Editorial Policies and the Editorial Policy Checklist.

Statistics

For all statistical analyses, confirm that the following items are present in the figure legend, table legend, main text, or Methods section.

n/a	Confirmed	
x	☐	The exact sample size (*n*) for each experimental group/condition, given as a discrete number and unit of measurement
x	☐	A statement on whether measurements were taken from distinct samples or whether the same sample was measured repeatedly
x	☐	The statistical test(s) used AND whether they are one- or two-sided<br>*Only common tests should be described solely by name; describe more complex techniques in the Methods section.*
x	☐	A description of all covariates tested
x	☐	A description of any assumptions or corrections, such as tests of normality and adjustment for multiple comparisons
x	☐	A full description of the statistical parameters including central tendency (e.g. means) or other basic estimates (e.g. regression coefficient) AND variation (e.g. standard deviation) or associated estimates of uncertainty (e.g. confidence intervals)
x	☐	For null hypothesis testing, the test statistic (e.g. *F*, *t*, *r*) with confidence intervals, effect sizes, degrees of freedom and *P* value noted<br>*Give P values as exact values whenever suitable.*
x	☐	For Bayesian analysis, information on the choice of priors and Markov chain Monte Carlo settings
x	☐	For hierarchical and complex designs, identification of the appropriate level for tests and full reporting of outcomes
x	☐	Estimates of effect sizes (e.g. Cohen's *d*, Pearson's *r*), indicating how they were calculated

Our web collection on statistics for biologists contains articles on many of the points above.

Software and code

Policy information about availability of computer code

Data collection	Data collected for Excitation Emission Matrices (EEMs) were collected in the Myneni and Onstott Labs at Princeton University. This data is presented in spectra format in Fig. 2 and Fig. S2
Data analysis	Fluorescence data files collected for Excitation-Emission Matrices presented in this manuscript were processed according to the publicly available "PARAFAC analysis of EEM data to separate DOM components in R" tutorial by Dr. Matthias Pucher (https://cran.r-project.org/web/packages/staRdom/vignettes/PARAFAC_analysis_of_EEM.html). For data processing presented in our manuscript, the version v.1.1.1 of the staRdom package, and version v.4.0.0 of RStudio were used to complete this analysis. Figure 2 was generated with the use of RStudio v.4.0.0 and the packages ggplot2 v.3.3.2 and reshape2 v.1.4.4. Figures 1,4,5, and 6 were generated in Excel v.16.70. Figure 3 was generated from negative ion ESI FT-ICR MS peak identifications using the PetroOrg software v.18.0.6 from the National High Magnetic Field Lab (https://nationalmaglab.org/user-facilities/icr/software/).

For manuscripts utilizing custom algorithms or software that are central to the research but not yet described in published literature, software must be made available to editors and reviewers. We strongly encourage code deposition in a community repository (e.g. GitHub). See the Nature Portfolio guidelines for submitting code & software for further information.

Data

Policy information about availability of data

All manuscripts must include a data availability statement. This statement should provide the following information, where applicable:

- Accession codes, unique identifiers, or web links for publicly available datasets
- A description of any restrictions on data availability
- For clinical datasets or third party data, please ensure that the statement adheres to our policy

The data generated in this study are provided in the Supplementary Information and Supplementary Data.

Human research participants

Policy information about studies involving human research participants and Sex and Gender in Research.

Reporting on sex and gender	*Use the terms sex (biological attribute) and gender (shaped by social and cultural circumstances) carefully in order to avoid confusing both terms. Indicate if findings apply to only one sex or gender; describe whether sex and gender were considered in study design whether sex and/or gender was determined based on self-reporting or assigned and methods used. Provide in the source data disaggregated sex and gender data where this information has been collected, and consent has been obtained for sharing of individual-level data; provide overall numbers in this Reporting Summary. Please state if this information has not been collected. Report sex- and gender-based analyses where performed, justify reasons for lack of sex- and gender-based analysis.*
Population characteristics	*Describe the covariate-relevant population characteristics of the human research participants (e.g. age, genotypic information, past and current diagnosis and treatment categories). If you filled out the behavioural & social sciences study design questions and have nothing to add here, write "See above."*
Recruitment	*Describe how participants were recruited. Outline any potential self-selection bias or other biases that may be present and how these are likely to impact results.*
Ethics oversight	*Identify the organization(s) that approved the study protocol.*

Note that full information on the approval of the study protocol must also be provided in the manuscript.

Field-specific reporting

Please select the one below that is the best fit for your research. If you are not sure, read the appropriate sections before making your selection.

☐ Life sciences ☐ Behavioural & social sciences ☒ Ecological, evolutionary & environmental sciences

For a reference copy of the document with all sections, see nature.com/documents/nr-reporting-summary-flat.pdf

Ecological, evolutionary & environmental sciences study design

All studies must disclose on these points even when the disclosure is negative.

Study description	This study includes qualitative and quantitative characterization of dissolved organic carbon in subsurface fracture fluids. Specific techniques included in the main text of this study are: 1) DOC/DIC quantification including measurements of low molecular weight organic acids, 2) d13C, 3) D14C, 4) Excitation-Emission Matrix Analysis, 5) Negative Ion ESI 21 Tesla FT-ICR MS, 6) semi-quantitative gas chromatography, 7) d2H and d13C of C1-C3 hydrocarbon species, and 8) HPLC detection of aspartic acid D/L ratio. Analyses 1 and 2 included three replicates for each sample. Analyses 3, 4, 5, 6, and 8 included a single measurement for each sample. Analysis 7 included two replicates for each sample.
Research sample	This study presents original quantification and characterization of the dissolved organic carbon (DOC), along with input from dissolved inorganic carbon (DIC) from two brine systems (95 and 101 level), one dolomitic fluid (1200 level), one gold-reef rock sample (Vaal Reef), and potential drilling contaminants (including mine service water), from one mine named Moab Khotsong. All fluids sampled from Moab Khotsong are naturally occurring fluids trapped within the mine host rock that were first intersected by mine personnel during exploratory drilling campaigns. New boreholes were drilled for subsequent research sampling. The mine service water originates as a mixture of 1200 level dolomitic fluid and meteoric water (including meteoric water run-off from mine tailings). The Vaal Reef sample represents DOC extracted from a piece of the main mine ore body collected by the mine and provided to South African collaborators following instructed sterile collection and handling direction. Fluid volume and replicate analysis from each site are constrained by the amount of fluid a given aquifer will emit during a sampling event (can range from a few liters to several hundred), the amount of fluid that can be carried out of the mine by research personnel, and the amount of time permitted by the mine for sample collection (4 hours maximum per day). Previous published characterization on these fluid systems indicates a low biomass microbial community in each, believed to be dominated by halophilic bacteria and archaea. It is not confirmed if the 101 level and 95 level brine systems are connected at some point throughout the mine as it is difficult to constrain the full extent of each fracture system.

Sampling strategy	All sample sizes and replicates included in this study are typical of subsurface fracture water sampling studies in which sampling event conditions and availability of fluid constrain sample material. All analytical methods presented in this manuscript are based on previously published protocols with defined thresholds of reproducibility and sample replicates.
Data collection	All fracture fluid and gas samples were collected from previously drilled boreholes at each of the three fluid sampling levels (95, 101, and 1200 levels). Collection of fluid at the 95-level occurred via an Inconel U-tube and packer device installed in 2018; the Inconel sampling line was flushed for several minutes prior to sample collection, and N2 was used to drive fluid from the sampling line prior to being sealed following sample collection. On the 101 and 1200 levels, a sterile, stainless steel multi-port manifold was attached to the borehole and fluid was flushed for several minutes prior to sample collection. The 101 level was sealed with a stainless-steel valve in between sampling events. The 1200 level was not sealed, but instead, fluid at the 1200-level continuously flows out of an uncapped rubber hose at a rate of ~15 L/min. Gas samples were collected from fluids with gas-stripping device. Data for fluid and gas flow rates, temperature, pH, ORP, and dissolved O2 were written down in a field notebook on-site by either Devan Nisson, Tom Kieft, Julio Castillo, or Oliver Warr at the time of collection. These geochemical and physiochemical parameters were previously published in the reference Nisson et al., 2023 and are not explicitly focused on in this study.
Timing and spatial scale	All samples were collected either during August 2018, August 2019, or January 2020 sampling trips. The August 2019 sampling trip was the longest duration (six weeks) and the others were two weeks in length. During each sampling trip, samples were collected from 2-4 days of the week in 4 hour underground shifts. All samples for DOC/DIC analysis were immediately placed on ice upon collection underground and transported to a freezer on surface. Samples were transported to Princeton university within a month using a liquid N2 charged dry shipper, and fluids were stored immediately at -20C. Samples for GC and volatile hydrocarbon isotope analysis were shipped within two months to Exxon Mobil and University of Toronto for immediate measurement, respectively. All samples were processed within 5 months of collection with the exception of fluids for FT-ICRMS and D14, which were processed within two years following sample collection. The potential influence of air leakage through cracked sealant grease into D14C DIC samples was noted in Appendix II.
Data exclusions	Measurements of d13C DIC were obtained from the Picarro CRDS at Princeton University (similar to the DOC d13C measurements presented in this manuscript) but the d13C DIC of the 101 level was from a 2018 sample in which the fluid collection had significant air exchange. Since there was no other 101 level brine from the 2019 or 2020 trips available for a secondary analysis, we chose to only present d13C DIC measurements from NOSAMS. Picarro d13C DIC values for the 95 and 1200 levels were within 2 per mil uncertainty to NOSAM measurements, leading to our confidence in reporting these NOSAM values for DIC.
Reproducibility	Reproducibility for DOC/DIC quantification were ±1% RSD based on triplicate measurements and d13C(DOC) analysis were ±0.3‰ V-PDB as run on triplicate measurements. For d13C DIC this was ±0.2‰ V-PDB for triplicate standard measurements. For aspartic acid racemization, up to 2.0% increase in D/L ratio was corrected for according to triplicate runs of a liquid aspartic acid standard. Following the methods of reference Ward et al., 2004, reproducibility was ±0.5‰ with respect to V-PDB standard for d13C of volatile hydrocarbons, and ±5‰ with respect to V-SMOW for d2H of volatile hydrocarbons based on duplicate measurements. Reproducibility for low molecular weight organic acid detection was ±10%RSD based on triplicate measurements. EEMs and UV-Vis analyses are presented based on one replicate sample. All attempts to repeat measurement of semi-quantitative methods EEMs, UvVis and volatile GC profile were successful in producing near identical spectra. FTICRMS profiles capture variability between potential sample replicates as all sample was combined in the SPE extraction and final sample eluent used for analysis.
Randomization	Randomization does not apply to this study, as samples considered for analysis were constrained by the ability to go underground for sample collection, and the quantity/ability of fluid to be emitted from each borehole on a given sampling trip and the quantity of fluid able to be carried above ground and transported to appropriate labs for subsequent analyses.
Blinding	Blinding was not relevant to this study as sample results were not influenced by research personnel understanding all details of sample collection, storage and other applicable characteristics.

Did the study involve field work? ☒ Yes ☐ No

Field work, collection and transport

Field conditions	Fluid sampling occurred at one borehole/level of the mine per day, and sampling events were limited to four hour windows underground. Boreholes on the 95 and 101 levels were sealed prior to and following sampling events, using an N2 flushed inconel U-tube sampling device (95 level) or sealed stainless steel valve (101 level). This is with the exception of the 2018 expedition in which the 101 level borehole was left unsealed, and allowed to exchange with air, and the use of 2019 and later samples is detailed in the methods of this manuscript for this reason. Ambient temperatures in the mine fluctuated around 30 degrees Celsius, and in response, all samples were placed on ice underground and quickly transported to surface (within an hour) after sampling and placed in proper refrigeration or freezer storage on surface at the mine. Facilities at the University of the Free State, Bloemfontein were used to sterilize equipment before application in the field (specifically, autoclave, oven, ethanol, and methanol).
Location	The location of all original samples [resented in this study was Moab Khotsong Mine, located in the Klerksdorp Mining District (26.98°S,26.78°E) of the Witwatersrand Basin in South Africa. Fluids were sampled from boreholes at 1.2km, 2.9km, and 3.1km depth in the mine, corresponding to mine levels of "1200 Level", "95 Level", and "101 Level", respectively. Sampling occurred in either August 2018, August 2019, or January 2020 sampling trips, with the most samples collected during the six-week campaign in 2019 (other trips were two weeks in length).
Access & import/export	Sampling events were coordinated with Moab Khotsong mine management to allow up to four external research members to go underground with proper personal protective equipment. This included visiting research members undergoing mine-specific safety training for use of emergency safety equipment. All materials and equipment were logged with the mine management upon entering

	and exiting the campus. A Precious Metals Special Permit was issued to and used by the University of the Witwatersrand to ship a section of the Vaal Reef to Devan Nisson at Princeton University (Issuing authority: Chief Executive Officer Cecil Khosa of S.A. Diamond and Precious Metals Regulator, Valid 05 May 2021 to 05 May 2022).
Disturbance	No disturbance was caused by this study.

Reporting for specific materials, systems and methods

We require information from authors about some types of materials, experimental systems and methods used in many studies. Here, indicate whether each material, system or method listed is relevant to your study. If you are not sure if a list item applies to your research, read the appropriate section before selecting a response.

Materials & experimental systems

n/a	Involved in the study
☒	☐ Antibodies
☒	☐ Eukaryotic cell lines
☒	☐ Palaeontology and archaeology
☒	☐ Animals and other organisms
☒	☐ Clinical data
☒	☐ Dual use research of concern

Methods

n/a	Involved in the study
☒	☐ ChIP-seq
☒	☐ Flow cytometry
☒	☐ MRI-based neuroimaging
